# Annotations

**Published:** 1896-09-19

**Authors:** 


### Annotations.

### The Treatment of Criminal Drunkards.

There appears to be only one thing in the way of the more scientific treatment of drunkenness in this country, and that is the fear lest the proposed compulsory detention of drunkards should be abused. This, however, is a very far-reaching and serious objection, and, in view of the outcry so frequently raised against our asylum system in regard to lunatics, it is difficult to believe that the public would accept any such proposal in regard to ordinary drunkards. The question is somewhat different, however, when applied to what one may call criminal drunkards. These are people who break the law, maybe only in small degree, but who still are lawbreakers, and are such almost exclusively in consequence of their excess in the use of alcohol. What Dr. Norman Kerr and those with him ask for is that, letting the non-criminal drunkard alone for the present, the criminal drunkard at least should be subjected to compulsory treatment for what may charitably be looked upon as his disease, be made to work, be made to keep off drink, be made to lead a sober, decent life, and only after sufficient time has elapsed to engrain upon him the habit of sobriety, to let him out again to try his fortune amid the temptations of the outer world. What the law now practically says to the drunken brawler is, "Take your punishment; then go and sin again!" What Dr. Norman Kerr would say to him is, "After your punishment is over we will keep you out of mischief for a time, accustom you to work and temperance, and make a good citizen of you once again." Ah, yes! If it can be done!

### Our Tobacco Pouches.

Far back in the very beginning of Bible history is the question, "Am I my brother's keeper?" a question which still, after the lapse of five or six thousand years, remains the only excuse that can be offered by those who, for the satisfaction of their daily wants, connive at the injury, and, in too many cases, the death, of others. The annual report of the Chief Inspector of Factories is full of examples of the dangerous risks to which workpeople are exposed in the manufacture of articles, the convenience of which we all appreciate. Miss Anderson, one of the inspectors reporting on the indiarubber works which she had visited in the Manchester district, in which bisulphide of carbon and naphtha are used, says that "even if, as stated, the use of bisulphide has decreased, there are certain processes in which it remains an essential part, *e.g.*, making of air balloons, tobacco pouches. . . and, in fact, all kinds of articles in which a high degree of elasticity is required. In all these cases I have found women and young girls at work, and in only one case have I found precautions systematically adopted which appear, so far, to have adequately protected them from the otherwise necessarily injurious effects of the fumes to which they are exposed." Now, what are the effects produced by this pleasing compound, the use of which is so necessary for the production of the "high degree of elasticity" required in our tobacco pouches. The mother of one girl says that she never expects to see her daughter the same girl again; that "she sits down in a stupor, or extremely drowsy condition, in front of the fire whenever she comes home, refusing food, and that also frequently she can only be got to bed by being carried there, while, if aroused, she gets wild and excited." All these symptoms, together with others, such as uncertain gait in walking, are attributed by Dr. Bury, in his work on peripheral neuritis to poisoning by bisulphide of carbon. Mr. Rogers, another of the inspectors, says, "most of the workers who are much exposed to these fumes complain of violent headaches, dizziness, and nausea, and in some instances a paralysis (peripheral neuritis) is set up. In one of the places I visited I found two workers who had apparently suffered from this disease recently; both had completely lost the use of their limbs for some weeks, and gradually recovered when they were not exposed to the fumes." In France women, and persons of both sexes under eighteen years of age, are excluded from employment in india-rubber works where such noxious fumes are given off; and in Belgium persons under sixteen are similarly excluded, by a recent decree, while women, and young persons between sixteen and twenty-one years of age, may not be employed for more than five hours a day, nor for more than two hours and a-half at one time. These are facts for smokers worthy of all consideration.

### Margarine and Deception.

Some of the most curious facts elicited by the recent Select Committee on Adulteration had reference to the trade in margarine. Properly prepared margarine is a perfectly healthy article of diet. It may not be quite as nice as good butter, but it is far better, and nicer also, than a great deal of the butter in the market; yet every effort is made to palm it off as butter, and not only so, but it appears that even people who know well what they are buying, who have not the slightest objection to margarine—in fact, want it—still dislike to have it labelled as such, or even to ask for it under its proper name. The report says: "The desire of purchasers to conceal from other customers the fact that they buy this article instead of butter induces them to abstain from asking for margarine under that name, and to demand butter at a low price." Such is pride! A sort of new language is invented, and it is recognized and understood that a request for butter at a certain price means margarine. And so to save the feelings of the British housewife the shopkeeper lays himself open to a charge under the Margarine Acts. There can be no doubt, however, that a large amount of fraudulent substitution of margarine for butter goes on. It appears that it is the custom to put up margarine in boxes and other receptacles of a shape similar to those in which butter is sent to market, and that every known form of butter package is imitated by the manufacturers of margarine, although considerable expense in addition to the increased cost of carriage is thereby involved; and the only object of all this must be to facilitate its fraudulent substitution for butter. It seems a pity that the trade in a perfectly wholesome food like margarine should be so surrounded by deceit that even the buyers, if not cheated, like to cheat themselves and pretend to be buying butter. How is the law to protect people who not only submit to adulteration but are parties to the fraud?

